# First report of a leopard (*Panthera pardus*)–bonobo (*Pan paniscus*) encounter at the LuiKotale study site, Democratic Republic of the Congo

**DOI:** 10.1007/s10329-021-00897-8

**Published:** 2021-05-05

**Authors:** Nicolas Corredor-Ospina, Melodie Kreyer, Giulia Rossi, Gottfried Hohmann, Barbara Fruth

**Affiliations:** 1 LuiKotale Bonobo Project, Centre for Research and Conservation, Royal Zoological Society of Antwerp, Antwerp, Belgium; 2 SEC Semillero de Evolución Y Conservación, Universidad Militar Nueva Granada, Cajica, Colombia; 3 Faculty of Science, School of Biological and Environmental Sciences, Liverpool John Moores University, Liverpool, UK; 4 Department of Human Evolution, Max Planck Institute for Evolutionary Anthropology, Leipzig, Germany; 5 Department for the Ecology of Animal Societies, Max Planck Institute for Animal Behavior, Konstanz, Germany

**Keywords:** *Pan paniscus*, *Panthera pardus*, Predation, Mobbing, Anti-predator strategies

## Abstract

Predation is a major cause of mortality in non-human primates, and considered a selective force in the evolution of primate societies. Although larger body size is considered as protection against predation, evidence for predation on great apes by carnivores comes from chimpanzees (*Pan troglodytes*), gorillas (*Gorilla gorilla*), and orangutans (*Pongo* spp.). Here, we describe the first encounter between wild bonobos (*Pan paniscus*) and a leopard (*Panthera pardus*). A single leopard was confronted by a group of habituated bonobos for three hours. Two adult males and one adolescent female bonobo actively harassed the leopard, which remained still for most of the encounter and reacted only to close approaches by bonobos. While no predation was observed, their behaviours confirm that bonobos perceive leopards as potential predators. Our report adds novel information to descriptions from other African ape species, and sheds light on the behavioural repertoire of bonobos’ anti-predation strategies. For future investigations, we suggest tagging leopards to remotely monitor their movements and allow assessment of encounter rates as one of several factors influencing predation pressure.

**Supplementary Information:**

The online version contains supplementary material available at 10.1007/s10329-021-00897-8.

## Introduction

Predation is widely considered a major selective force in the evolution of primate social systems (van Schaik and Hörstermann 1994) and sociality (Cheney and Wrangham 1987). Evidence suggests that predation impacts primate species’ behaviour and survival despite the wide variations in gregariousness, spatial proximity among group members, group size and composition, and habitat use (Isbell 1994; Zuberbühler 2007). Although predation pressure may decrease with increasing body size of prey species, field reports show that even the largest species are vulnerable to attacks by predators (Klailova et al. 2012).

Encounters between chimpanzees (*Pan troglodytes*) and leopards (*Panthera pardus*) have been observed at various field sites from West (Zuberbühler and Jenny 2002) to East Africa (Nishie 2018; Pierce 2009), and in some populations leopard predation was considered responsible for almost 40% of adult mortality cases (Boesch 1991). In addition to direct observations, indirect evidence of predation events comes from procedures such as scat or bone inspections (Eller et al. 2020; Nakazawa 2020). In Taï (Côte d’Ivoire), chimpanzees have been shown to respond to predation pressure by decreasing party sizes while increasing the sex ratio of parties to include more males, although overall party size was larger than in East African chimpanzee sites with lower predation pressure (Boesch 1991). When leopards are detected nearby, chimpanzees sometimes mob, chase and even deprive them of their prey (Hiraiwa-Hasegawa et al. 1986; Nakamura et al. 2019; Nishie 2018). Compared to chimpanzees, little is known about other ape species’ reactions in encounters with large carnivores. Remains of gorillas (*Gorilla gorilla*) have been found in leopard scats (Hayward et al. 2006) and reports of encounters between gorillas and leopards confirm that the latter elicit escape or defensive behaviours (see review by Klailova et al. (2012)). There are no observations of predation by carnivores on orangutans (*Pongo* spp.) but indirect evidence suggests that attacks by clouded leopards (*Neofelis nebulosa*) may occur (Knott et al. 2019).

Despite the evidence of immature and adult apes’ vulnerability to non-human predation, intensity of predation pressure (i.e. the rate of encounter with predators), and anti-predation strategies remain understudied. One obstacle to obtaining such information is human observers. Although behavioural studies of wild great apes rely on habituation to observers, human presence reduces encounters with predators and lowers the probability of attacks, leading to underestimates of predation pressure (but see Isbell and Young (1993)). In fact, faecal analyses revealed that hunting of chimpanzees and gorillas by leopards was related to ape abundance (Hayward et al. 2006; Henschel et al. 2011). Recently, remote sensing technology was used to monitor the behaviour of prey species (vervet monkeys *Chlorocebus pygerythrus* and baboons *Papio anubis*) and leopards, to obtain unbiased information on the relationship between primates and large carnivores (Isbell et al. 2018). However, such methods are not always feasible, particularly in a dense forest environment with closed cover, and where researchers still rely on alternative ways to get information. Potential anti-predator behaviours can be useful for estimating the significance of predation in a given species or population. For example, great ape nest site choice has been shown to reflect variation in predation pressure (Fruth and Hohmann 1996; Fruth et al. 2018) with chimpanzees at a predator rich site building their nests higher and in less accessible spots than where predators are absent (Pruetz et al. 2008; Stewart and Pruetz 2013). Baboons have been observed to change their sleeping sites after leopard predation events (Matsumoto-Oda 2015).

Leopard predation may also affect grouping patterns (Boesch 1991) and travel decisions (Janmaat et al. 2014). Considering the behavioural response of prey species in the presence of predators, Dutour et al. (2016) proposed that mobbing predators posed a relatively high low risk. Thus, anti-predator behavioural strategies may serve as an indirect reference for prey-predator relationships. One way to explore responses to predators is field experiments that expose apes to potential natural risks (Girard-Buttoz et al. 2020; Kortlandt 1962). A more common source of information comes from direct observation of natural encounters with predators.

Here, we present observational data from an encounter between a group of habituated bonobos and a male leopard at the field site of LuiKotale, Democratic Republic of the Congo. To our knowledge, this is the first report on a direct encounter between wild bonobos and a large carnivore. The only other information comes from the same population and concerns remains of an immature bonobo obtained from leopard faeces (D'Amour et al. 2006). In bonobo habitat—the dense lowland forests south of the Congo river—lions (*Panthera leo*), hyenas (*Crocuta crocuta*), and wild dogs *(Lycaon pictus*) are absent, whereas golden cats (*Caracal aurata*), crested hawk eagles (*Stephanoaetus coronatus*) and pythons (*Python sp.*) occur and may be considered as potential predators, in addition to leopards (Kano 1992). Although not predators, other snakes such as vipers (*Bitis spp.*) or cobras (*Naja spp.*) can be considered as potentially deadly threats. Bonobo death rates attributable to the above-mentioned species are probably low compared to those caused by humans due to poaching, habitat alteration, population growth and migration (Fruth et al. 2016). Our report contributes to the knowledge about natural predator–prey interactions by offering a descriptive account of an encounter between members of a party of bonobos and one leopard.

## Study site and species

The LuiKotale camp (2°45′S, 20°22′E) borders the Salonga National Park in the Democratic Republic of the Congo (Fruth and Hohmann 2018). The LuiKotale Bonobo Project (LKBP) started in 2002 and has maintained a permanent presence of researchers ever since. The mammalian fauna in the study area is representative of the central Congo basin (Bessone et al. 2020; Campbell 2005), including nine species of diurnal non-human primates (*Piliocolobus tholloni, Colobus angolensis, Lophocebus aterrimus, Cercocebus chrysogaster, Allenopithecus nigroviridis, Cercopithecus ascanius, C. mona wolfi, C. neglectus*), bonobo (*Pan paniscus*), forest elephant (*Loxodonta africana cyclotis*), red river hog (*Potamochoerus porcus*), bongo (*Tragelaphus eurycerus*), sitatunga (*T. spekii*) and several species of forest duikers (*Cephalophus silvicultor, C. dorsalis, C. nigrifrons, C. weynsi, Philantomba monticola*).

Currently, the LKBP monitors three bonobo communities: two habituated communities, namely the Bompusa West (WBp) fully habituated since 2007 and the Bompusa East (EBp) fully habituated since 2015; and the Lombo (Ekongo) community, currently undergoing habituation (Fruth and Hohmann 2018). Ranges of the three communities partially overlap and are similar in terms of vegetation cover and forest structure. The WBp community contained a total of 52 individuals at the time of the event (32 adults and 20 immatures).

## Evidence of the presence of leopards in the study area

Leopards and golden cats are the large carnivores occurring in the area. Although both species are rarely seen, previous studies (D'Amour et al. 2006), occasional sightings by research assistants, ad libitum camera trap footage and records from a systematic survey (Bessone et al. 2020) confirm their continuous presence across the entire study site and adjacent forests.

## Observations on July 17th 2020

**Fig. 1:**
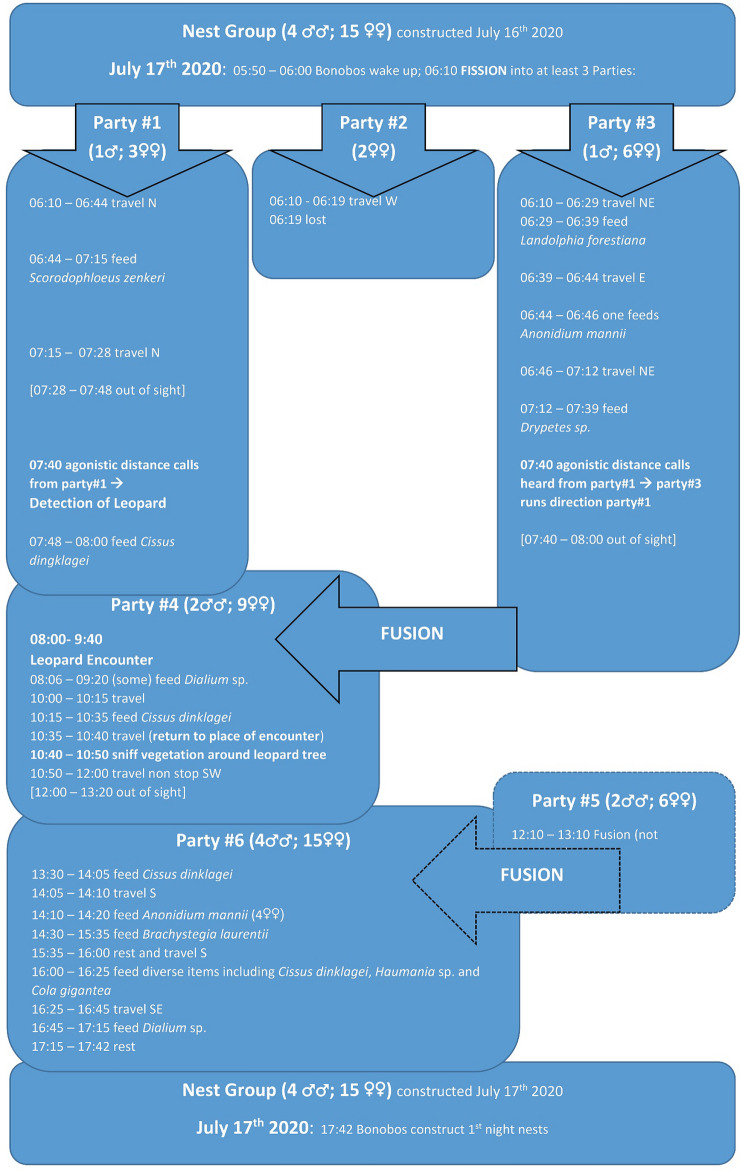
Fission–fusion pattern and activities of parties and the encounter of the Bonobo Bompusa West community with a leopard, July 17th 2020, LuiKotale, DRC

*05:25*: Mélodie Kreyer (MK), Giulia Rossi (GR), and Nicolas Corredor-Ospina (NC) start observations at a nest site of the WBp community. The night-nest party is composed of 19 mature individuals, four males (one adolescent; three adult); and 15 females (five nulliparous; nine with offspring; one old) (see Fig. 1 for a schematic depiction of the day).

*06:10*: Individuals descend to the ground and split into several smaller parties that move in different directions:

### Party #1 followed by NC: consisting of one adult male and three females (two nulliparous; one with offspring)

*06:44*: After splitting from the rest of the nest group, party #1 travels north. Bonobos climb a tree and feed on pods of *Scorodophloeus zenkeri* for 30 min before descending and continuing north.

*07:48*: After hearing distance vocalizations at around 07:42, NC catches up with the party. Bonobos are up in the trees giving agonistic calls (type contest hoots, screams and whistle-barks sensu De Waal (1988)). While approaching the group, NC notices a group of roughly ten red river hogs foraging on the shrub *Cissus dinklagei*, and mistakenly attributes the bonobos’ calls as reaction to the river hogs. However, in response to an unidentified roaring from the canopy, the hogs flee (07:53), while the bonobos continue emitting agonistic calls. With this roaring later attributed to a leopard, bonobos were estimated to be in proximity to the leopard since 07:40.

### Party #2 followed by GR: consisting of two adult females with offspring.

*06:15*: After splitting from the rest of the nest group, party #2 travels west towards the Badzungu river, where they are lost from view just before crossing the river (06:19). GR returns and joins MK with party #3 (06:30).

### Party #3 followed by MK and GR: consisting of one adult male and six females (two nulliparous; three with offspring; one old)

*06:20*: After splitting from the rest of the nest group, party #3 moves north-east and feeds on *Landolphia forestiana* for less than 10 min. After a brief stop during which the adult male fed on *Anonidium mannii,* the group travelled about 2 km north-east and entered another feeding tree (*Drypetes* sp.) at 07:12, where they fed until 07:39.

*07:40*: MK and GR hear distance calls from party #1, which cause some members of party #3 to appear nervous and agitated. All individuals descend rapidly and start rushing in the direction of the vocalizations of party #1. GR and MK follow the direction of the distance calls, briefly losing sight of party #3.

*08:00*: NC observes fusion of party #3 with party #1, resulting in party #4. MK and GR arrive (08:05). Individuals of party #1 did not change their behaviour after fusion with party #3.

### Party #4 followed by MK, GR, and NC: consisting of two adult males and nine females (four nulliparous; four with offspring; one old).

*08:05*: Focusing on the arboreal source of roaring vocalizations, observers locate a leopard, surrounded by party #4 members in neighbouring trees. The leopard’s body is hidden by what looks like the remains of a bonobo nest, with only the tail visible from the ground. The nest is on branches about 10–12 m high, in the middle of a *Dialium* sp. tree.

*08:06*: Emil, an adult male bonobo, is in a nearby tree, close (4–5 m) to the leopard and sporadically shaking branches. The rest of the party is in the canopy, surrounding the leopard’s tree from a distance of 10–20 m. Some of the adults continue vocalizing loudly towards the leopard. A few feed on *Dialium* leaves, but in general all look highly alert.

*08:19*: The leopard appears nervous, frantically swaying its tail and roaring loudly in response to bonobos’ intermittent agonistic vocalizations and displays. However, the leopard does not move from his arboreal spot for over an hour.

*09:13*: Emil descends to the ground, followed by other individuals. Most individuals, including two adult females and infants inspect the ground and smell what seems to be leopard pee. The nulliparous female Flora remains in the leopard’s tree and approaches “his” nest.

*09:19*: Flora, about 5 m away from the nest is threatened away by the leopard. This is the first aggression towards a bonobo. The other individuals respond by hitting tree trunks with hand and feet and by vocalizing loudly. Emil and others climb up trees again.

*09:20*: Jack, an adult male, climbs the leopard’s *Dialium* tree, stopping to smell the trunk where claw marks are visible, and the surrounding vegetation. An unidentified individual climbs the tree and approaches to 5–7 m from the nest. At this point the leopard jumps from the nest and chases the approaching bonobo away. The leopard stops at the main trunk, looks at the observing humans, and returns to the nest. Bonobos continue shaking branches, hitting trunks, and screaming at the leopard. At least three nulliparous, one old, and two adult females with their offspring remain on the ground while both adult males, the other nulliparous and two adult females with their offspring are up in the trees. The leopard roars and moves towards any bonobo that approaches to within 5 m. Emil, Jack and Flora are most active in harassing the leopard from near this distance. Most infants remain close to their mothers apart from one male who actively inspects the ground and returns to his mother at each new sound from above. Whenever the leopard leaves the nest and roars to try to displace the arboreal harassers, terrestrial bonobos respond by jumping back into trees and joining in the chorus of screams and barks. All party members give agonistic vocalizations; three of them stay close without actively harassing the leopard, while three (Emil, Jack, Flora) get closest to it. Others, such as Paula, a high-ranking female, stays in the leopard’s tree and feeds on *Dialium* leaves.

*09:40*: A total of nine of the 11 mature bonobos of party #4 start slowly and silently to withdraw from the area. Two, Flora and Jack, remain in the tree and continue approaching the leopard in its nest.

*09:43*: Jack moves nearer, and displaces the leopard, which roars and moves towards Jack before jumping to a higher spot. Immediately, Jack inspects the inside of the empty nest, smelling it. Jack then descends to the ground, leaving Flora alone in the tree.

*09:45*: Flora continues harassing the leopard, until it jumps and chases her away. In response, bonobos again climb nearby trees, scream and shake branches. This time the leopard retreats to the *Dialium’s* tree highest point, when Jack again ascends the tree and returns to the nest, which he sniffs. Flora again approaches the leopard to within 4–5 m, hitting her support-branch with her feet and hands, and flailing an arm towards the leopard. The leopard shows its teeth in a clear threat (Video 1).

*09:49*: A female with offspring joins the party.

*10:00*: All bonobos descend and walk around 100 m to feed on *Cissus dinklagei*. Researchers follow the bonobos, losing sight of the leopard, still in the tree.

*10:25*: The two males, and six females (three nulliparous; three adult) descend from their arboreal food patch and return to the *Dialium* tree where the leopard was earlier; the other bonobos continue feeding. The returning individuals climb up, sniff the vegetation in and around the nest, and leave 5 min later. The leopard was not seen or heard again.

*10:50*: Bonobos leave the area and walk rapidly back south-west, neither foraging nor engaging in any sort social interaction. They cross the Badzungu river and move into the most western part of their home range. They travel almost continuously until party #4 fuses with other members (party #5) of the community at 12:00–13:20, feeding on *C. dinklagei* fruits, about 4 km in a beeline from the location of the leopard encounter. During the rest of the day the fused party #6 (containing four males and 16 females), continues travelling, feeding, and resting, until they build their night nests starting at 17:42 (Fig. 1).

The leopard was a male, probably not fully grown. No injuries or signs of disability were noted. There was no physical contact between bonobos and the leopard. Immature bonobos remained in physical contact with their mothers.

## Discussion

This report of an encounter between wild bonobos and a leopard demonstrates that bonobos at LuiKotale perceive leopards as a potential predator. Considering body size and physical force, leopards are certainly the most dangerous nonhuman predator for bonobos. Adult bonobos weigh between 24 and 43 kg (Grawunder et al. 2018) and by that fall within the range of preferred prey species (Henschel et al. 2011). Although the leopard did not show any intention to capture a bonobo, the encounter supports earlier evidence confirming its role as bonobo predator (D'Amour et al. 2006). While this single observation does not permit any conclusion, it sheds light on components of the behavioural ecology of bonobos, specifically in the context of predator avoidance. Given that field research on wild bonobos started in the late 1970s, it is remarkable that this is the first report describing an encounter. Possible explanations for the lack of observations are: (i) study sites are located in areas of low leopard density; (ii) the presence of human observers deters leopards; and (iii) bonobos avoid close encounters with leopards.

(i) At LuiKotale, leopards are seen occasionally by researchers. Camera trap evidence confirms the species’ presence and its activity during both daytime and nighttime (*own unpublished data*), suggesting that terrestrial encounters with bonobos are a likely scenario. During daily follows of habituated bonobos it is not uncommon to hear high pitched agonistic hoots and screams without being able to detect what caused this behaviour. Therefore, although reliable estimates of encounter rates between bonobos, leopards, and other potentially dangerous animals remain unavailable, it is likely that bonobos encounter leopards more often when traveling without human observers. Systematic studies on leopard behavioural ecology, prey species selection, and hunting strategies offer an exciting approach to complement long-term studies on habituated great apes.
(ii) Although LuiKotale leopards are not habituated to human observers, it is unlikely that human presence significantly influenced the encounter described here, including how long the leopard stayed. The leopard returned repeatedly to the nest after lunging out displacing bonobos probably because bonobos seemed to have blocked potential arboreal escape routes, as well as the area below the tree. Nevertheless, we cannot exclude the possibility that the leopard was distracted by humans and prevented from launching more serious attacks.
(iii) Tagging leopards to monitor their movements and behaviour would allow comparisons of predator and prey ranging patterns, and help to understand bonobos’ grouping patterns, travel decisions, and communication in response to leopard ranging and predation. Although this seems like a feasible alternative to the multiple concerns about tagging great apes (Jenny and Zuberbühler 2005), it requires thorough ethical consideration. Until we obtain insights from complementary studies, we have to rely on inferring avoidance and approaches from behavioural responses. The initial response that alerted us to the encounter was in the form of distant vocalisations inciting reunification of two smaller parties that had previously split, enlarging the party to 11 mature and four immature individuals, a strategy in line with proposed advantages of group living (e.g. dilution effect: (Delm 1990)). In response to potential predators apes may flee, charge, or engage in mobbing (Klailova et al. 2012). Individuals engaging in mobbing behaviour are exposed to the risk of physical damage, and differential engagement in mobbing indicates individual variation in risk taking (Crofoot 2012; 2013; Micheletta et al. 2012). In non-human primates, risk taking is an important parameter of personality and differs across individuals and species (Freeman and Gosling 2010). Comparative studies of captive and wild apes suggest that bonobos tend to avoid risky strategies more than do chimpanzees (Haun et al. 2011; Kalan et al. 2019). Based on our observations, bonobos differed widely in their behavioural response. All individuals approached the tree where the leopard was and emitted at least some agonistic calls, most remained at least 10 m from it, However, the dominant female of the group ate in the same tree where the leopard was resting, and only three individuals actively harassed the leopard, provoking it to leave his resting place and charge the nearest individuals. These were two adult males whose mothers were dead, and a recently immigrated adolescent female. Adult males without a mother have a relatively low status within the community’s hierarchy, as have immigrant females (Surbeck et al. 2011; Toda and Furuichi 2020). Immigrant females tend to seek proximity to senior, high-ranking females with whom to form associations (Furuichi 1989; Idani 1991; Sakamaki et al. 2015). As only the highest-ranking female ate calmly in the same tree where the leopard rested, Flora may have sought her attention by showing off in the same tree, while the two males may have used the opportunity to try to impress Flora, as their chances of mating with Flora are not increased by the presence of a mother (Furuichi 1997; Surbeck et al. 2011). While the three individuals’ behaviour might appear particularly risky, it may have been facilitated by the nearby, vocally mobbing larger group providing a combination of safety in numbers, dilution, predator confusion, and potential communal defence (Curio 1978; Hamilton 1971).

An alternative interpretation focuses on curiosity. Kalan et al. (2019) showed that bonobos exhibit more curiosity than chimpanzees and gorillas. The bonobos we observed showed frequent sniffing and visual inspection both arboreally and on the ground, suggesting that such close leopard encounters are not an everyday event. Hosaka and Ihobe (2015) reported that chimpanzees also sniffed leaves and footprints that likely had traces of a leopard’s odour, suggesting similar degrees of predator-elicited curiosity in bonobos and chimpanzees.

Although previous studies have reported greater neophobia and risk avoidance in bonobos compared to chimpanzees (Herrmann et al. 2011; Kalan et al. 2019), the behaviours of the LuiKotale bonobos suggest a spectrum of responses rather than a direct match between the two species. For example, responses including aggressive behaviours such as screaming, shaking branches, and approaching a potential predator have been reported in Mahale chimpanzees (Hiraiwa-Hasegawa et al. 1986). Similarly, bonobos moved away silently after the first encounter, fed, then returned slowly and silently to inspect the area of the encounter, a behaviour sequence also observed in Mahale (Nishie 2018). So, we believe it is important to note greater similarities in bonobos’ and chimpanzees’ reactions towards a predator than previously expected. Further behavioural studies at different sites may contribute more useful observations on anti-predator strategies within and across species.

## Supplementary Information

Below is the link to the electronic supplementary material.

Supplementary file1 (MP4 39227 KB)
